# Does psychological functioning mediate the relationship between bullying involvement and weight loss preoccupation in adolescents? A two-stage cross-sectional study

**DOI:** 10.1186/s12966-017-0491-1

**Published:** 2017-03-24

**Authors:** Kirsty Lee, Alexa Guy, Jeremy Dale, Dieter Wolke

**Affiliations:** 1 Department of Psychology, University of Warwick, Coventry, CV4 7AL UK; 2 Warwick Medical School, University of Warwick, Coventry, CV4 7AL UK

**Keywords:** Weight loss preoccupation, Adolescents, Bullying, Victimisation, Psychological functioning

## Abstract

**Background:**

Adolescent bullying is associated with a range of adversities for those who are bullied i.e., victims and bully-victims (e.g., those who bully others and get victimised), including reduced psychological functioning and eating disorder symptoms. Bullies are generally well-adjusted psychologically, but previous research suggests that bullies may also engage in problematic diet behaviours. This study investigates a) whether adolescents involved in bullying (bullies, victims, bully-victims) are at increased risk of weight loss preoccupation, b) whether psychological functioning mediates this relationship and c) whether sex is a key moderator.

**Method:**

A two-stage design was used. In stage 1, adolescents (*n* = 2782) from five UK secondary schools were screened for bullying involvement using self and peer reports. In stage 2, a sample of bullies, victims, bully-victims and uninvolved adolescents (*n* = 767) completed a battery of assessments. The measures included the eating behaviours component of the Child and Adolescent Psychiatric Assessment, which was reduced to one factor (weight loss preoccupation) and used as the outcome variable. Measures of self-esteem, body-esteem and emotional problems were reduced to a latent (mediator) variable of psychological functioning. Multi-group analysis examined the effects of sex and all models were adjusted for covariates (BMI, pubertal stage, age, parental education and ethnicity).

**Results:**

Bullies, victims and bully-victims were at increased risk of weight loss preoccupation compared to adolescents uninvolved in bullying. The mechanism by which bullying involvement related to increased weight loss preoccupation varied by bullying role: in bullies the effect was direct, in victims the effect was indirect (via reduced psychological functioning) and in bully-victims the effect was both direct and indirect. Sex significantly moderated the relationship in bullies: weight loss preoccupation was only statistically significant in bullies who were boys.

**Conclusion:**

Bullying involvement during adolescence is associated with weight loss preoccupation. Bullies are likely driven by a desire to increase attractiveness and social status; whereas weight loss preoccupation in bullied adolescents may have maladaptive influences on diet and exercise behaviours due to its association with reduced psychological functioning. Future research should consider peer victimisation as a potential modifiable risk factor for reduced psychological functioning and weight loss preoccupation, which if targeted, may help to prevent maladaptive diet and exercise behaviours.

**Electronic supplementary material:**

The online version of this article (doi:10.1186/s12966-017-0491-1) contains supplementary material, which is available to authorized users.

## Background

Bullying, defined as the intentional and repeated harm caused by peers where there is a real or perceived power imbalance [1], is pervasive [2, 3]. Bullied adolescents, i.e., victims and bully-victims (those who bully others and get bullied themselves), experience wide-ranging and long-lasting adverse effects on their psychological and psychiatric health, such as low self-esteem [4], depression [5], psychosis [6] and self-harm [7].

Bullying can be physical (e.g., hitting, kicking), relational (e.g., spreading rumours in person or online) or verbal (e.g., name calling). It is well documented that being bullied verbally, particularly about appearance, can negatively affect body-esteem (i.e., body image) and lead to disordered eating [8]. There is emerging evidence that any type of peer victimisation (e.g., physical, relational, cyber) can have similar adverse effects on body-esteem [9] and diet behaviours [10] in victims and bully-victims.

Bullies, those who perpetrate bullying and are never victimised, also appear to be at increased risk of eating disorder symptoms [10, 11]. This is noteworthy because bullies tend to be well-adjusted psychologically and suffer few negative consequences as a result of harming others [12]. Bullying is principally a means to achieve status and access to resources [13]. Research suggests that bullies are bi-strategic, in that, to obtain dominance in the peer group they reduce the status of their victim through aggressive acts [14, 15] and use self-promotion to enhance their own desirability [16]. During adolescence, attractiveness is a highly valued status characteristic [17] and this is often represented as a slim and curvaceous ideal for females [18] and a slim and muscular ideal for males [19]. Obtaining either of these ideals may require a significant amount of weight control through diet and exercise. Research to date has not investigated the extent to which bullies, victims and bully-victims are preoccupied with losing weight.

If bullies, victims and bully-victims are all at increased risk of weight loss preoccupation, is it via the same pathways? Copeland and colleagues found that increased emotional problems was the mechanism by which bullying involvement led to eating disorder symptoms [10], which is not surprising considering the comprehensive effects that being bullied has on emotional problems and psychological functioning [20] (such as self-esteem and body-esteem). However, bullies tend to have good psychological functioning [21], are often popular in the peer group and enjoy high social status [22]. It is thus plausible that psychological functioning may play a mediating role between bullying involvement and preoccupation with weight loss in victims and bully-victims, but not bullies.

Reduced psychological functioning may mediate the relationship between bullying involvement and a preoccupation with controlling weight, but there are potentially moderating factors in addition. Research indicates that girls are at greater risk of disordered eating and body dissatisfaction [23], whilst boys are more likely to engage in eating and exercise strategies to build muscle or lose weight [24, 25]; because of such differences in the potential strategies used by males and females, sex should thus be examined as a potential moderator. Other factors that can influence body-image and weight control behaviours are body mass index, pubertal stage, age, ethnicity and socioeconomic status [23, 26–30]. Identifying such mediating and moderating factors could help to guide clinicians and aid in the targeting of interventions for bullied adolescents.

This study investigates whether bullies, victims and bully-victims are at increased risk of weight loss preoccupation compared to adolescents uninvolved in bullying, whether psychological functioning mediates the relationship between bullying role and weight loss preoccupation, and whether sex is a key moderator.

## Methods

### Design and sample

A power analysis was conducted based on research indicating that 100 participants per group (e.g., victims, uninvolved) are sufficient to detect moderate differences in body image [9]. Bullies have the lowest self-reported prevalence rate (2-5%) [21, 31] so were used as the lead group. A minimum of 2500 pupils needed to be screened to obtain 100 bullies. However, attrition in school-based studies occurs at a rate of around 30%, thus an initial sample of 3250 was needed.

A two stage sampling approach was used. In Stage 1, secondary school pupils (aged 11–16) were screened for bullying involvement using self-report and peer nominations. All those who screened positive for bullying others (i.e., bullies) were invited to take part in Stage 2, alongside a random selection of victims, bully-victims and adolescents uninvolved in bullying. Pupils from each school who completed stage 1 and 2 were entered into a prize draw to win a £50 voucher.

**Fig. 1:**
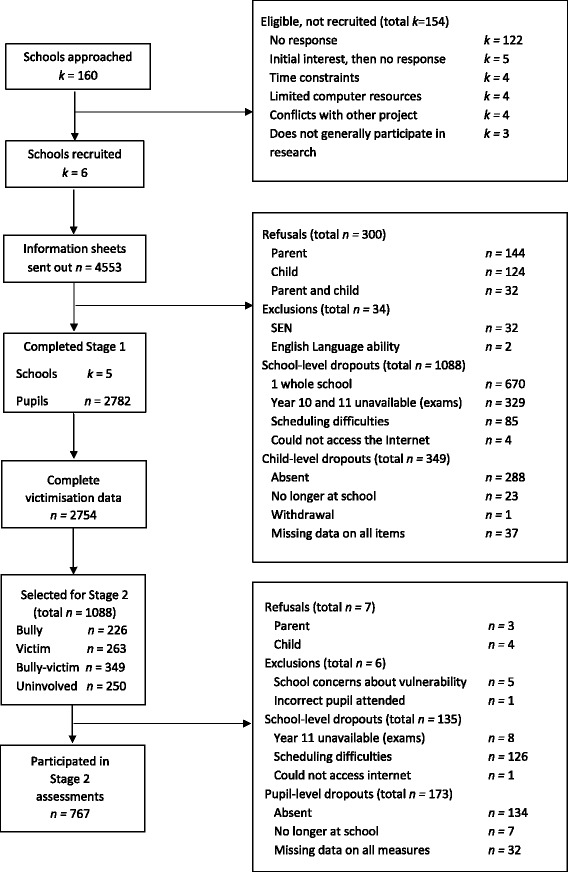
STROBE flow diagram of recruitment and selection of schools and pupils

School recruitment took place between July 2014 and February 2015 and data collection took place between September 2014 and July 2015. Head teachers of secondary schools in the UK were approached with full details of the study (k = 160) (Fig. 1). Five schools (mixed sex *n* = 4; single sex [girls] *n* = 1) agreed to participate in the study. All pupils (*n* = 3883) were invited to participate in The Bullying, Appearance, Social Information Processing and Emotions Study (The BASE study). However, at no point was the term bullying used (pupils were invited to take part in a "Relationships, Health and Emotions" study). Written information sheets were sent home in sealed envelopes. Parents were asked to return an opt-out form if they did not want their child to participate. As shown in the STROBE diagram [32] 2782 (71%) pupils provided informed written consent and were screened for bullying involvement (Fig. 1). Decision rules to assign screened pupils to the potential bullying roles are shown in Table 1. As there were a large number of pupils who were victims, bully-victims or uninvolved in bullying, a sub-selection balanced by sex were selected using Microsoft Excel’s random number generator. In total, 1088 pupils were selected for Stage 2.

**Table 1:** Rules used to assign adolescents to a bullying role for the Stage 2 assessments

Role	Rule
Bully	Self-reported bully OR peer nominated bully (z-score >1) AND not a self-reported or peer nominated victim.
Victim	Self-reported victim (several times a week) AND not a self-reported or peer nominated bully (z-score <1).
Bully-victim	Self-reported bully and victim OR peer nominated bully (z-score >1) and victim (z-score >1) OR any combination self-reported or peer nominated bully and victim.
Uninvolved	Not a self-reported victim or bully AND no peer nominations as a victim or bully.

In Stage 2, 306 of the selected pupils were absent from school or could not take part due to school organisational difficulties (i.e., one school was unable to allocate the maximum time and computer resources needed for the study). Three parents refused their child’s participation (bully *n* = 1, uninvolved *n* = 2), four pupils refused to participate (bully-victims *n* = 4) and five were excluded due to school concerns about vulnerability (victim *n* = 1, bully-victim *n* = 3, uninvolved *n* = 1). In total 767 pupils had data on the outcome measure (weight loss preoccupation). Just over half of the sample (52.9%) were female and the mean age was 13.6 years (SD = 1.4).

### Measures

Electronic questionnaires were completed in a school IT lab or classroom on a PC, laptop or tablet, with at least one investigator present. Bullying and demographic information were obtained at Stage 1 and the remaining measures were assessed at Stage 2, approximately 2 months later.

### Bullying role (predictor)

Bullying role was assessed at Stage 1 using self-report and peer nominations. *Self-reported bullying* was based on the Bullying and Friendship Interview schedule [33], a validated measure of bullying behaviour [34, 35]. The scale included 13 behavioural descriptions and assessed three different types of bullying, i.e., direct (e.g., “been hit or beaten up”), relational (e.g., “had lies/nasty things spread about you”) and cyber (e.g. “had embarrassing pictures posted online without permission”). The same items were repeated with slight wording adaptations to assess bullying perpetration. Pupils were asked how frequently any of these behaviours had occurred during the past 6 months with responses of never, sometimes, quite a lot (several times a month) or a lot (at least once a week). Response of “quite a lot” or “a lot” indicated bullying involvement [33, 35].

For the *peer nominations*, pupils were given a list of names of all the peers in their form/tutor group (e.g., Homeroom or Registration group) and asked to nominate up to three pupils (not themselves) who were victims or perpetrators of bullying behaviours (e.g., “Some people are repeatedly hit, shoved around, beaten up, threatened, blackmailed, insulted, called nasty names, played tricks on or stolen from. Which people in your form/tutor group have these things happened to?”). Z-scores were created using the total number of nominations received per pupil within each tutor group. Pupils were identified as involved in bullying if their z-score was one standard deviation above the tutor group mean on the bullying item (bullies), victimisation item (victims) or on both items (bully-victims). Pupils were identified as uninvolved if they received zero nominations on the bullying and victimisation items.

### Individual characteristics (covariates)

Sex, age, ethnicity and parent education (a proxy for socioeconomic status) were self-reported at Stage 1. Ethnicity was dummy coded as White British or Other, as there were too few participants in each ethnic category to allow meaningful comparisons (e.g., the next largest ethnic group was Asian at 6.1%). Parent’s highest level of education i.e., did not complete school (<11 years), basic schooling (11 years), college (11–13 years) or university (>13 years), was dummy coded into 0 = 13 years or less (≤13) and 1 = more than 13 years (>13) of education.

Pubertal development was assessed at stage 2 using the Pubertal development scale (PDS) [36].

The validity of the PDS has been assessed by comparing self-reported development with physician ratings of Tanner Stages (i.e., the gold standard test) [37]. Correlations between the PDS and physician rated Tanner Stage range between *r =* .61 and *r =* .67, suggesting the PDS is an adequate indicator of pubertal maturation. Cronbach alphas in the current study were acceptable for girls (α = .67) and boys (α = .75). In females, ratings of body hair growth, breast development and menarche were assessed; in males, ratings of body hair growth, voice change and facial hair growth were assessed. Scale scores were transformed into five pubertal (Tanner) stages [37]. The stages were on a five-point scale (1 to 5), with higher stages indicating more advanced development.

Height and weight were measured at stage 2. Weight was measured to the nearest 0.1 kilogram using Tanita BC-1000 portable electronic scale (Tanita Corporation, Tokyo, Japan), whilst wearing lightweight clothes with shoes and jackets removed. Height was measured to the nearest 0.1 centimetre using a portable stadiometer (Leicester height measure, Child Growth Foundation, UK). Body mass index (BMI) was calculated by dividing weight in kilograms by height in meters squared (kg/m^2^) and was subsequently converted into a percentile score using international BMI for age and sex cut-offs [38]: percentile scores ranged between 1 (<3rd percentile; severely underweight) and 5 (>97th percentile; obese).

### Psychological functioning (mediator)

Pupils completed Rosenberg’s Self-Esteem Scale [39] and the Strengths and Difficulties Questionnaire (SDQ) [40] at Stage 1 and the Body Esteem Scale for Adolescents and Adults [41] at Stage 2, which are well-validated scales that have been used in numerous studies of adolescence [42–46]. Rosenberg’s Self-Esteem Scale is a 10-item scale, responded to on a 4-point scale (0 = strongly agree; 3 = strongly disagree), with higher scores indicating higher self-esteem (Cronbach α = .89). The Body Esteem Scale for adolescents and adults is a 23-item scale, responded to on a 5-point scale (0 = never; 4 = always), with higher scores indicating higher body-esteem (α = .93). The SDQ is a 25-item scale consisting of five factors: hyperactivity-inattention, emotional problems, peer problems, conduct problems, and prosocial behaviour. For the purpose of this study only the emotional problems subscale was used (5-items). Responses were on a 3-point scale (0 = not true; 2 = certainly true) and higher scores indicated higher emotional problems. For consistency with the self-esteem and body-esteem scores, the emotional problems score was reverse coded, so that higher scores indicated fewer emotional problems (and higher esteem) (α = .75). Total scores on the self-esteem, body-esteem and emotional problems scales were used to generate a composite (latent) variable of psychological functioning, whereby higher scores indicated higher psychological functioning and wellbeing.

### Weight loss preoccupation (outcome)

At Stage 2 pupils completed an adapted version of the eating behaviours component of the Child and Adolescent Psychiatric Assessment (CAPA) version 5.0 [47]. The CAPA is an interview schedule that has been used to diagnose a variety of psychiatric illnesses in children and adolescents, including eating disorders [10]. The first adaptation was to make the questions suitable for self-completion, rather than interviewer led. Other adaptations include the rewording of items (e.g., “are you afraid of getting fat?” to “are you afraid of putting on weight?”) and the inclusion of associated items (e.g., “are you afraid of losing weight?”). The items used in the current study are reported in Table 3 (see table and footnote). Responses were on a three-point scale (0 = never; 2 = often), except for one item on dieting (“have you ever dieted?” response of “no” or “yes”) and one item on weighing frequency (response of “once or more a day”, “once or more a week”, “once or more a month”, “hardly ever/never”). Responses to all items were subsequently dummy coded (0 = no, 1 = yes).

### Analysis

**Fig. 2:**
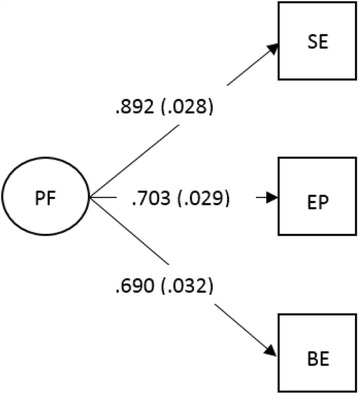
Factor loadings (with standard errors in parenthesis) of self-esteem (SE), emotional problems (EP) and body-esteem (BE) onto the latent psychological functioning (PF) variable

**Fig. 3:**
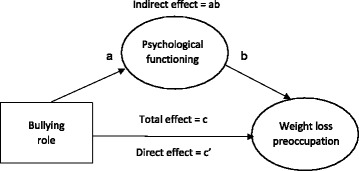
Simplified hypothetical mediation model showing the direct, indirect and total effects. *Note*: The total effect (c) is the effect of bullying role on weight and exercise concern with the inclusion of psychological functioning. The direct effect (c’) is the effect of bullying role on weight loss preoccupation without the inclusion of psychological functioning. The indirect effect (ab) is the effect of bullying role on weight loss preoccupation, via psychological functioning

All analyses were performed using Stata 14. Missing and descriptive data were analysed (Table 2). Structural equation models were then built up sequentially. Firstly, exploratory factor analysis was performed on the 18 items of the adapted CAPA, using principal component factor analysis and a loading value of ≥ .40 for item inclusion (Table 3): the resulting 7-item factor – weight loss preoccupation – was calculated into a (weighted) factor score and was used as the latent outcome variable in all subsequent analyses. Secondly, confirmatory factor analysis was performed on the total scores of the self-esteem, body-esteem and emotional problems scales, which were used as indicators of psychological functioning (latent mediator variable) (see Fig. 2). Thirdly, recursive structural models were built; that is, we specified predictive links from bullying role to weight loss preoccupation, which included an indirect path via psychological functioning (see Fig. 3 for a hypothetical model). Using the uninvolved group as the reference category, dummy variables were created for each bullying role (e.g., uninvolved = 0, victim = 1) and models were computed for each bullying role separately to examine the unadjusted direct effect of bullying role on weight loss preoccupation, and the indirect (mediated) effect via psychological functioning (Table 4, model 1). We then adjusted the models for sex and the covariates (BMI, pubertal stage, age, parental education and ethnicity) (Table 4, model 2); modification indices (i.e., the Lagrange Multiplier test) were used to estimate which parameters should be included to improve model fits [48] (Table 4, model 3). Lastly, we used multi-group models to test the potential moderating effect of sex. Model fits were assessed using the root-mean square error of approximation (RMSEA), Comparative Fit Index (CFI) and Tucker-Lewis index (TLI) indices: RMSEA values less than 0.06 and CFI and TLI values greater than 0.95 indicate a close (i.e., good) fitting mode, though RMSEA values less than 0.08 and CFI and TLI values greater than 0.90 are acceptable [49–51]. Full information maximum likelihood estimation was used in all modelling to account for missing data. All model estimates are expressed as standardised regression coefficients (β).

**Table 2:** Descriptive data and group differences for each bullying role, presented as percentages, means and standard deviation (M ± SD)

	*N*	Uninvolved	Bully	Victim	Bully-victim	*p*
*N*	767	174	150	140	303	
%		22.7	19.6	18.3	39.5	
Sex (%)						
*Girls*	406	50.6	51.3	67.9	48.2	*.001*
*Boys*	361	49.4	48.7	32.1	51.8	
Ethnicity (%)						
*White British*	648	84.4	81.2	87.0	86.1	.48
*Other*	115	15.6	18.8	13.0	13.9	
Parent education (%)						
*< 13 years*	536	65.5	70.7	71.4	71.3	.56
*> 13 years*	231	34.5	29.3	28.6	28.7	
Age *(M ± SD)*	767	13.5 ± 1.4	13.9 ± 1.4	13.6 ± 1.4	13.6 ± 1.3	.10
Pubertal stage *(M ± SD)*	570	2.5 ± 0.8	2.7 ± 0.7	2.6 ± 0.8	2.6 ± 0.7	.23
BMI percentile *(M ± SD)*	367	3.2 ± 0.7	3.1 ± 0.9	3.2 ± 0.9	3.3 ± 0.9	.20
Psychological functioning *(M ± SD)*	521	0.5 ± 0.8	0.3 ± 0.8	−0.6 ± 0.9	−0.2 ± 1.0	*.001*
Weight loss preoccupation *(M ± SD)*	521	−0.4 ± 0.9	−0.0 ± 1.0	0.1 ± 1.0	0.2 ± 1.0	*001*

**Table 3:** Relationships among loadings, communalities and factor reliability (α) for girls, boys and the total sample on weight loss preoccupation

	Factor: Weight loss preoccupation	Communalities
Items^a^		
Trying to lose weight	.70	.51
Worried about putting on weight	.64	.44
Exercises to lose weight	.61	.41
Worries about food	.53	.34
Dieted to lose weight	.46	.24
Eaten less	.46	.23
Worries if cannot exercise	.41	.23
Cronbach α (total)	.76	
Girls	.78	
Boys	.73	

^a^ Excluded items were: Lost weight; Eaten more; Put weight on; Self-weighs frequently; Exercises for muscle; Trying to stay the same weight; Trying to gain weight; Fasted to lose weight; Vomited or taken laxatives; Taken diet pills or powders

**Table 4:** Fit indices (FI) for each specified model for bullies, victims and bully-victims

Role	FI	MODEL		
		Model 1^a^	Model 2^b^	Model 3^c^
Bully	CFI	.813	.716	.944
	TLI	.625	.562	.895
	RMSEA	.168	.117	.057
Victim	CFI	.924	.866	.963
	TLI	.848	.793	.930
	RMSEA	.148	.101	.058
Bully-Victim	CFI	.882	.800	.981
	TLI	.763	.691	.964
	RMSEA	.166	.114	.039

^a^Model 1 was the unadjusted model i.e., Fig. 3

^b^Model 2 was adjusted for sex, BMI, pubertal stage, age, parental education and ethnicity; i.e., direct paths between each variable and weight loss preoccupation were included

^c^The Lagrange Multiplier test was used to estimate which parameters should be included to improve model fits. Additional parameters included in model 3 were indirect paths between sex, pubertal stage and BMI percentile on weight loss preoccupation via psychological functioning and error covariance between body-esteem and weight loss preoccupation

## Results

### Missing and descriptive data

Missing data on the outcome variable (weight loss preoccupation) were not related to bullying role, BMI percentile, sex, ethnicity, parent education, age, pubertal stage, body-esteem or emotional problems, but was related to self-esteem; adolescents with higher self-esteem had lower odds of missing data (OR = 0.90, 95% CI = 0.83 to 0.99, *p* = .024). Overall, missing data were highest on BMI percentile (41.4%), body-esteem (30.4%) and pubertal stage (25.8%). BMI data were missing mostly due to school time constraints (*n* = 278) or refusals (*n* = 82); we speculate that missing data were high on body-esteem and pubertal stage due to the sensitive nature of such questions.

Descriptive data for each bullying role are reported in Table 2. The majority of the sample were bully-victims (39.5%) and victims were mostly girls (67.9%). There were no significant differences between bullies, victims, bully-victims and uninvolved adolescents on any of the covariates.

### Exploratory factor analysis

The Kaiser-Meyer-Olkin measure of sampling adequacy was .67 and Bartlett’s test of sphericity was significant (*χ* ^2^ (153) = 1210.38, *p* < .001), indicating the minimum standards for conducting factor analysis were met. Eleven items were excluded (see footnote of Table 3) and one factor with seven items was extracted (eigenvalue = 2.15) and identified as weight loss preoccupation. Factor loadings, ordered by size of loading, communalities, and factor reliability are shown in Table 3.

### Confirmatory factor analysis (psychological functioning)

Because all possible coefficients were estimated the model was saturated (RMSEA = 0.000, CFI = 1.000, TLI = 1.000): these fit indices do not represent a perfect, nor a problematic model [52]. Factor loadings were high (Fig. 2), suggesting that high self-esteem, body-esteem and few emotional problems were strong indicators of psychological functioning.

### Structural model

A hypothetical (unadjusted) model is displayed in Fig. 3. The fit indices for this model (Table 4, model 1) were poor for bullies, victims and bully-victims.

In model 2 (Table 4), paths were adjusted for sex, BMI, pubertal stage, age, parental education and ethnicity; i.e., direct paths between each variable and weight loss preoccupation were included. Fit indices were reduced further when covariates were included into the model.

In model 3 (Table 4), modification indices were used to test for the statistical significance of omitted paths. Additional paths were included if the modification index was substantial or the path was theoretically justifiable [53]; we included indirect paths between sex, pubertal stage and BMI percentile on weight loss preoccupation via psychological functioning. Previous research indicates that girls, adolescents with early-onset advanced pubertal stage and adolescents with obesity are at increased risk of depression, low self-esteem and poor body image [54–56], meaning these paths were theoretically plausible. In the current study, girls (*M* = −.37, *SD* = .98) had significantly (*p* < .001) poorer psychological functioning than boys (*M* = .43, *SD* = .83) and there were significant negative correlations between psychological functioning and pubertal stage (*r* = −.13, *p* = .007) and between psychological functioning and BMI percentile (*r* = −.21, *p* < .001). An additional parameter was included to allow for error covariance between body-esteem and weight loss preoccupation. Including these additional parameters produced an acceptable fitting model for bullies and good fitting models for victims and bully-victims (Table 4, model 3). The path estimates of the final model (i.e., model 3) for bullies, victims and bully-victims are reported in Table 5. Path estimates of the covariates are reported in Additional file 1.

**Table 5:** Standardised regression coefficients (β) and standard errors in parenthesis (SE) of the total, direct and indirect effect of weight loss preoccupation in bullies, victims and bully-victims

	Bullying role					
	Bully		Victim		Bully-victim	
	β (SE)	*p*	β (SE)	*p*	β (SE)	*p*
Total effect (c)	**.218 (.113)**	*<.001*	**.260 (.123)**	*<.001*	**.292 (.102)**	*<.001*
Direct effect (c’)	**.183 (.110)**	*.001*	.141 (.153)	.076	**.179 (.115)**	*.001*
Indirect effect (ab)	.035 (.040)	.096	**.120 (.106)**	*.030*	**.114 (.069)**	*.001*

Note: Each bullying role was compared to the uninvolved group. All models controlled for sex, BMI percentile, pubertal stage, age, ethnicity, parent education, and included indirect paths between sex, pubertal stage and BMI percentile on weight loss preoccupation via psychological functioning and error covariance between body-esteem and weight loss preoccupation

Bullies, victims and bully-victims had increased weight loss preoccupation compared to adolescents uninvolved in bullying (i.e., total effects). In bullies, there was a significant direct relationship between being a bully and weight loss preoccupation; there was no evidence of mediation via psychological functioning. In victims, there was a significant indirect effect; that is, the relationship between victimisation and weight loss preoccupation was mediated by reduced psychological functioning. Bully-victims had characteristics of both bullies and victims as both the direct and indirect paths were significant, though the direct effect was stronger (β = .179) than the indirect effect (β = .114). Overall, bully-victims had the greatest weight loss preoccupation.

### Multi-group analysis

**Fig. 4:**
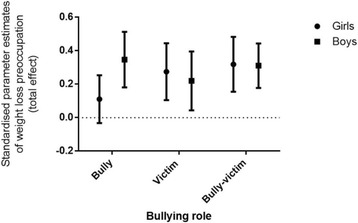
Standardised parameter estimates (β) with standard errors of the total effect of bullying role on weight loss preoccupation, stratified by sex. *Note*: The uninvolved group were used as the reference category at the zero line. Estimates were adjusted for BMI, pubertal stage, age, ethnicity and parent education

Total effects for bullies, victims and bully-victims, stratified by sex are displayed in Fig. 4. In bullies, the fit indices for the multi-group analysis were good (CFI = .939, TLI = .901, RMSEA = .049) and improved on the previous model fits (Table 4, model 3). There was evidence of moderation by sex on the parameter estimates; there was a strong direct effect of being a bully on weight loss preoccupation in boys (β = .316, SE = .144, *p* < .001) but not in girls (β = .078, SE = .157, *p* = .305). In contrast, the fit indices in victims (CFI = .925, TLI = .878, RMSEA = .072) and bully-victims (CFI = .938, TLI = .899, RMSEA = .061) were reduced in comparison to the previous model fits (Table 4, model 3); there was no evidence of moderation by sex on the relationship between being bullied and weight loss preoccupation. Moderation effects on the covariates are reported in Additional file 1.

## Discussion

This study found that adolescents involved in bullying in any role were at increased risk of weight loss preoccupation compared to adolescents uninvolved in bullying. There were distinct mechanisms for bullies, victims and bully-victims. Weight loss preoccupation in bullies was direct and unrelated to psychological functioning, whereas in victims the effect was mediated by reduced psychological functioning. Bully-victims had characteristics of both bullies and victims; weight loss preoccupation was directly related to bully-victim status and was partially mediated by reduced psychological functioning. The relationship between being a bully and weight loss preoccupation was moderated by sex: only bullies who were boys were preoccupied with losing weight.

A novel finding in this study was that bullies were almost equally likely as victims and bully-victims to be preoccupied with losing weight, which was unrelated to their psychological functioning. This supports research suggesting that bullies are psychologically well-adjusted but cool manipulators [12, 57]. We speculate that bullies may be preoccupied with controlling their weight as a strategy to achieve an ideal body type. This would fit with the theory of bullies striving for social dominance and access to resources (including romantic and sexual opportunities), whereby bullies attempt to enhance their own desirability whilst derogating their competitors [14–16]. These strategies appear to be fruitful, in that bullies have greater dating success [15], and adolescents who are both aggressive and have more peer-valued characteristics (like physical attractiveness and athletic capability) are more popular, powerful [22] and tend to have high levels of resources control [58].

Another novel finding was that weight loss preoccupation was only present in bullies who were boys. At first, this appears to conflict with research suggesting boys are under pressure and striving to be muscular [24, 59]. However, body-image in boys tends to have a U-shaped association, whereby body-satisfaction decreases the further away from ‘average’ boys perceive their body to be [60]. As teacher and self-reports suggest that male bullies are already physically strong [61, 62], male bullies may therefore be focussed on attaining or maintaining the slim-muscular ideal [19]. Male bullies tend to be more narcissistic [58] and there is some evidence of an association between narcissism and other-rated attractiveness [63]. It is unclear whether male narcissistic bullies are intrinsically more attractive, or they spend an increased amount of time and energy on their appearance.

Overall, girls had more weight loss preoccupation than boys, which is consistent with previous research [23, 64]. Surprisingly, girls who were bullies did not have increased weight loss preoccupation compared to adolescents uninvolved in bullying. In children [65] and adolescents [66], aggression and popularity are associated with peer and teacher nominated physical attractiveness. Females who aggress against their peers and are popular (i.e., pure bullies) may therefore perceive themselves as attractive and be less concerned about losing weight; although female bullies are inherently competitive, it is possible they do not endorse the thin-ideal [67].

Being bullied had comprehensive effects on psychological functioning, as has been found previously [20]. The findings here expand on previous research by suggesting that reduced psychological functioning, as a result of peer victimisation, may be driving weight loss preoccupation in victims. Both thin-body preoccupation [68, 69] and poor psychological functioning (i.e., depression and low self-esteem) [70] have been associated with pathological eating behaviours. Concerns about the body’s shape or size can also influence health on a physical and physiological level: both frequent and infrequent dieting can promote weight gain in girls and boys [71], and dissatisfaction with body size or shape can predict variation in inflammatory markers [72]. Peer victimisation can also act as a barrier to adolescents engaging in healthy physical activity [73]. Longitudinal research is needed to examine whether bullied adolescents are at additional risk of future health problems as a result of maladaptive diet and exercise cognitions and behaviours.

Bully-victims had characteristics of both bullies and victims. Like bullies, bully-victims had weight loss preoccupation irrespective of psychological functioning. This may similarly be explained by a desire to increase social status and romantic opportunities, especially as bully-victims are often considered to be unpopular and unattractive [74, 75]. However, like victims, weight loss preoccupation was also driven via reduced psychological functioning. Previous research has found that bully-victims are at the greatest risk of eating disorders [10, 11] and we found that overall bully-victims had the highest weight loss preoccupation. This adds to mounting evidence that bully-victims are at the greatest risk of multiple and adverse outcomes [11, 76]. It is thus important that bullying researchers consider bully-victims as a distinct group [77].

The strengths of this study include: a two-stage sampling process that identified all bullying roles (bullies, victims, bully-victims and uninvolved); a large sample of bullies, which can be difficult to obtain due to low prevalence (e.g., 2-5%) when using self-report measures [21, 31]; the use of validated measures of bullying, self-esteem, body-esteem and emotional problems; and a new measure of weight loss preoccupation validated through factor analysis.

There are some limitations to the study. Firstly, the weight loss preoccupation measure contained items relating to diet and exercise thoughts and behaviours; previous research suggests that eating disorder thoughts and behaviours are distinct factors, with the latter being the strongest predictor of depression [78]. However, an increased odds of eating disorder behaviours in adolescents involved in bullying has been found using the same instrument [10]. Secondly, few participants were non-White British, so there is uncertainty about the applicability of the findings to other ethnic groups. Previous research suggests those with White ethnicity are at the greatest risk of body dissatisfaction, disturbed eating [79] and victimisation [80, 81]. Thirdly, the schools involved in the study were from a relatively small geographical area in the UK, so the findings may not be generalisable beyond the current context. Finally, the cross-sectional design means that causality cannot be inferred, and researchers have warned that mediation analysis on cross-sectional data can be problematic [82]. However, studies of mono-zygotic twins have established that being bullied is a causal factor of reduced psychological functioning [20, 83], whilst meta-analysis and longitudinal studies suggest that self-evaluations of the body and its appearance is an established risk factor for problematic weight control behaviours [84–86]. Thus, bullying involvement and reduced psychological functioning as causal factors of weight loss preoccupation are plausible, but the findings require replication using a longitudinal design.

## Conclusions

In conclusion, bullying involvement during adolescence is a potentially modifiable environmental risk for weight loss preoccupation. In bullies, strategies to control weight are likely motivated by a desire for status and admiration; in victims, weight loss preoccupation is likely the result of peer victimisation, which adversely impacts psychological functioning. Bully-victims share characteristics of both bullies and victims and bullying researchers should consider bully-victims as a distinct group. Engaging in eating and exercise thoughts and behaviours to lose weight can have maladaptive influences on health, and experiencing peer victimisation should be considered as a potential risk factor. For clinicians and practitioners dealing with victims of peer bullying, therapies aimed at improving self-esteem, body-esteem and reducing emotional problems may prove beneficial for improving psychological wellbeing, and potentially inhibit the development of more widespread health problems, such as disordered diet and exercise behaviours.

## Additional file

Additional file 1: (DOCX 12 kb)

## Abbreviations

95% CI: 95% confidence interval
BMI: Body mass index
CAPA: Child and adolescent psychiatric assessment
CFI: Comparative fit index
FI: Fit indices
M: Mean
OR: Odds ratio
r: Pearson product–moment correlation coefficient
RMSEA: root-mean square error of approximation
SD: Standard deviation
SDQ: Strengths and difficulties questionnaire
TLI: Tucker-lewis index
α: alpha
β: beta
*χ*^2^: Chi-squared
