# Supplementary material for: Does psychological functioning mediate the relationship between bullying involvement and weight loss preoccupation in adolescents? A two-stage cross-sectional study
Source: Int J Behav Nutr Phys Act. 2017 Mar 24;14:38. doi: 10.1186/s12966-017-0491-1 (PMC5364676; doi:10.1186/s12966-017-0491-1)
Supplement: Additional file 1: — (DOCX 12 kb) [file 12966_2017_491_MOESM1_ESM.docx]

**Additional file 1**

**Bullies**

There were significant direct paths between BMI percentile (β=.221, SE=.095, p=.006) and parent education (β=.156, SE=.113, p=.004) on weight loss preoccupation; there were significant indirect paths of sex (β=-.154, SE=.097, p=.002) and BMI percentile (β=.087, SE=.040, p=.010) on weight loss preoccupation via reduced psychological functioning.

The direct path between parent education and weight loss preoccupation was significant in girls (β=.190, SE=.159, p=.011) and insignificant in boys (β=.077, SE=.148, p=.300). The indirect path of BMI percentile on weight loss preoccupation via reduced psychological functioning was significant in girls (β=.084, SE=.051, p=.030) and insignificant in boys (β=.099, SE=.063, p=.095).

**Bully-victims**

There were significant direct paths between BMI percentile (β=.142, SE=.085, p=.043), parent education (β=.093, SE=.101, p=.045) and age (β=-.137, SE=.045, p=.023) on weight loss preoccupation; there were significant indirect paths of sex (β=-.137, SE=.079, p<.001), BMI percentile (β=.058, SE=.027, p=.010) and pubertal stage (β=-.034, SE=.023, p=.046) on weight loss preoccupation via reduced psychological functioning.

The direct effect of BMI percentile on weight loss preoccupation was significant in bully-victims who were boys (β=.243, SE=.102, p=.010) and insignificant in girls (β=.070, SE=.163, p=.577); similarly, the direct effect of age on weight loss preoccupation was significant in bully-victims who were boys (β=-.178, SE=.065, p=.038) and insignificant in girls (β=-.117, SE=.068, p=.211). Similar to bullies, the indirect path of BMI percentile on weight loss preoccupation via reduced psychological functioning was significant in girls (β=.068, SE=.044, p=.046) and insignificant in boys (β=.046, SE=.029, p=.091). The indirect path of pubertal stage on weight loss preoccupation via reduced psychological functioning was significant in boys (β=-.061, SE=.037, p=.043) and insignificant in girls (β=-.022, SE=.032, p=.291).

**Victims**

There were significant direct paths between BMI percentile (β=.232, SE=.093, p=.002) and sex (β=-.164, SE=.150, p=.033) on weight loss preoccupation; there was a significant indirect path of sex on weight loss preoccupation via reduced psychological functioning (β=-.070, SE=.066, p=.039).

There was no evidence of moderation in victims.
